# Basal autophagy is pivotal for Hodgkin and Reed-Sternberg cells' survival and growth revealing a new strategy for Hodgkin lymphoma treatment

**DOI:** 10.18632/oncotarget.10300

**Published:** 2016-06-27

**Authors:** Katrin Birkenmeier, Katharina Moll, Sebastian Newrzela, Sylvia Hartmann, Stefan Dröse, Martin-Leo Hansmann

**Affiliations:** ^1^ Dr. Senckenberg Institute of Pathology, University Hospital of Frankfurt, 60596 Frankfurt am Main, Germany; ^2^ Clinic of Anesthesiology, Intensive-Care Medicine and Pain Therapy, Goethe-University Hospital, 60596 Frankfurt am Main, Germany

**Keywords:** B-cell lymphoma, Hodgkin lymphoma, autophagy, lymphoma pathogenesis, targeted therapy

## Abstract

As current classical Hodgkin lymphoma (cHL) treatment strategies have pronounced side-effects, specific inhibition of signaling pathways may offer novel strategies in cHL therapy. Basal autophagy, a regulated catabolic pathway to degrade cell's own components, is in cancer linked with both, tumor suppression or promotion. The finding that basal autophagy enhances tumor cell survival would thus lead to immediately testable strategies for novel therapies. Thus, we studied its contribution in cHL.

We found constitutive activation of autophagy in cHL cell lines and primary tissue. The expression of key autophagy-relevant proteins (e.g. Beclin-1, ULK1) and LC3 processing was increased in cHL cells, even in lymphoma cases. Consistently, cHL cells exhibited elevated numbers of autophagic vacuoles and intact autophagic flux. Autophagy inhibition with chloroquine or inactivation of ATG5 induced apoptosis and reduced proliferation of cHL cells. Chloroquine-mediated inhibition of basal autophagy significantly impaired HL growth *in-vivo* in NOD SCID γc^−/−^ (NSG) mice. We found that basal autophagy plays a pivotal role in sustaining mitochondrial function.

We conclude that cHL cells require basal autophagy for growth, survival and sustained metabolism making them sensitive to autophagy inhibition. This suggests basal autophagy as useful target for new strategies in cHL treatment.

## INTRODUCTION

With an incidence of about 3 new patients per 100.000 persons per year in Western countries, classical Hodgkin lymphoma (cHL) is one of the most frequent lymphomas. cHL is usually treated with radiation therapy, chemotherapy or hematopoietic stem cell transplantation [1–3]. However, current treatment strategies have pronounced side-effects, which arise as high risk acute and long-term toxicity including secondary neoplasia, organ toxicity to heart and lung, fatigue, and infertility. Thus, the current goal in the treatment of HL patients is to reduce toxicity but maintain efficacy [4–8]. In this regard, considering the dependency of Hodgkin lymphoma cells on multiple deregulated signaling pathways [9–14] targeted cHL therapy, e.g. specific inhibition of signaling pathways, may offer new strategies to improve cHL treatment.

As basal autophagy with its tumor-promoting and tumor-suppressing properties, it has become an attractive target for the development of novel therapeutic strategies in a series of solid tumors [15–23]. Basal autophagy allows the recycling of bioenergetic components [15–18]. In normal cells it promotes cell survival in response to nutrient deprivation [18–20]. In line with the contextual pro- and anti-survival effects in non-malignant cells, the role of basal autophagy in tumor cells is complex, depending on the tumor type and in part, on the stage of the disease [16, 17, 20–23]. Thus, it is critical to determine its contributions in one particular tumor.

A number of drugs that effectively inhibit basal autophagy are available, including chloroquine (CQ) and its derivatives [15, 16, 22–24]. So, the finding that basal autophagy enhances tumor cell survival would lead to immediately testable strategies for novel therapies in one particular tumor. Thus, we studied the role of basal autophagy in the pathogenesis of cHL.

## RESULTS

### Autophagy-relevant proteins are up-regulated in cHL

**Figure 1:**
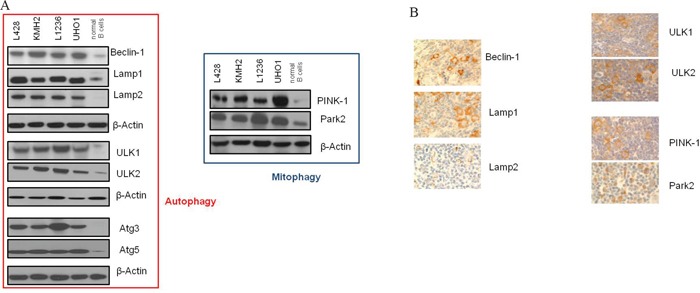
Autophagy-relevant genes are up-regulated in cHL **A.** Expression analysis by Western Blot was performed of a total of 9 autophagy-relevant proteins in cHL cell lines compared to normal non-malignant GC B cells. Shown is one representative Blot for each protein tested of a total of n=5 experiments. β-Actin was used as a loading control. **B.** Immunohistochemical stainings of paraffin sections of lymph nodes from patients with cHL for autophagy-relevant proteins (400x magnification).

Activated autophagy has been found to be linked in cancer with over-expression of autophagy-relevant proteins [29]. Therefore, we analyzed a set of key autophagy proteins by Western Blot in a panel of cHL cell lines and compared them to non-malignant GC B cells (Figure 1A, Supplemental Figure 1A). Quantification by Western Blot revealed an up-regulation of all 9 tested autophagy-relevant proteins in cHL cells. Among these were important regulators of autophagy process like Beclin-1, Lamp1, ATG5, and ULK1. We also observed high expression of Park2 and PINK-1, two regulatory proteins of mitophagy. In contrast, we found compared to GC B cells no up-regulated autophagy markers in BL and DLBCL cell lines (B cell non-Hodgkin lymphoma (B-NHL) cell lines) (Supplemental Figure 1B, 1C).

To validate these results in lymphoma primary tissue we additionally performed immunohistochemistry and compared the lymphoma staining to that of control tissue (normal non-malignant lymphoid tissue). We found more than 50% cHL cells positive for Beclin-1, Lamp1/2, PINK-1, Park2, and ULK1/2 in at least 12/17 cases (in 12/17, 15/17, 15/17, 14/17, 15/17, 13/17, and 16/17 cases). Reactive infiltrate was mostly negative in the tested cases. Germinal centers of control tissue, as well as B-NHL cases showed no detectable expression of the autophagy proteins tested in all analyzed samples (Figure 1B, Supplemental Table 4, Supplemental Figure 1D, 1E).

### cHL cells exhibit increased numbers of autophagosomes, high LC3II and intact autophagic flux

**Figure 2:**
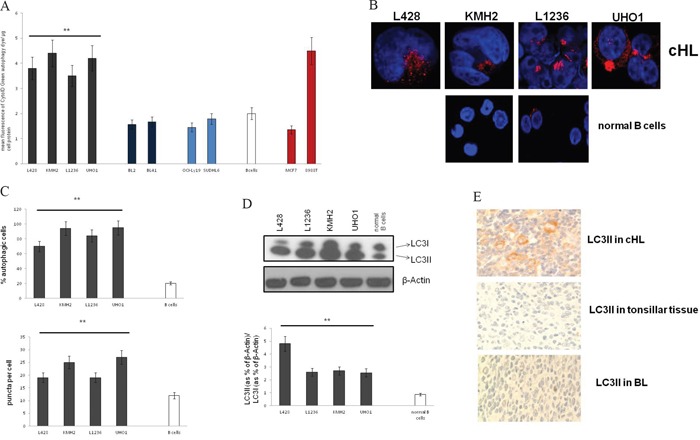
LC3 processing and the number of autophagosomes are increased in cHL **A.** Analysis of the mean fluorescence by FACS after staining of autophagosomes was performed in cHL, BL, and DLBCL cell lines, GC B cells, MCF7 cells and 8988T cells. Results are expressed as means±SD for n=6 experiments. ** cHL cell lines significantly different vs. GC B cells by the t-test (p<0.001). **B.** Fluorescence micrographs are shown of LC3II puncta in cHL cell lines and GC B cells. **C.** % of autophagic cells (*upper panel*), and LC3 puncta per cell (*lower panel*) were determined by confocal fluorescence microscopy of fixed cells stained with an antibody to LC3II. For quantification of autophagic cells pictures were taken under constant settings of 5-10 cells per micrograph. % of LC3II-positive cells was investigated of totally 50 cells per cell type. Fluorescent puncta were counted per cell of totally 50 cells per cell type and the mean value of puncta was calculated. Data are presented as means±SD. ** Cell lines significantly different vs. control group by the t-test (p<0.001). **D.** LC3 expression was analyzed by Western Blot in cHL cell lines and normal B cells (*upper panel*) and analyzed as the ratio of LC3II to LC3I (*lower panel*). Shown is one representative Western Blot of a total of n=5 experiments. Cells were grown under normal cell culture conditions, and lysates were prepared from cells that were no longer left in culture than 48 hrs without passaging to prevent autophagy-inducing nutrient deprivation. Densitometric analysis was performed using ImageJ. Results are expressed as means±SED; ** cHL cell lines significantly different from control group by the t-test (p<0.01). **E.** Immunohistochemical staining of cHL cases and normal non-malignant lymphoid tissue (control tissue) to LC3II.

Next, we quantified autophagosomes by using the Cyto-ID Green autophagy dye, which selectively stains autophagic vacuolar components [30]. Live-cell analysis by FACS revealed high fluorescence intensity of cHL cells of all 4 cHL cell lines analyzed (L428, KMH2, L1236, UHO1), indicating increased number of autophagic vacuoles in these cells, in contrast to the low fluorescence intensity of the BL cell lines BL2 and BL41 and DLBCL cell lines OCI-Ly19 and SUDHL6. Moreover, fluorescence intensity of the 4 cHL cell lines was comparable to that of the pancreatic cell line 8988T, which has recently been characterized as strongly autophagic [29] and was therefore used as positive control for high basal autophagy activation in these experiments. The low autophagic breast cancer cell line MCF7 [29] established as negative control showed the lowest fluorescence intensity of all cell types tested (Figure 2A).

To validate these data we performed immunofluorescence stainings with an antibody to LC3II (processed LC3), a standard method to measure basal autophagy [29, 31] (Figure 2B, 2C). Consistently, the number of LC3II puncta in cHL was high indicating at least 70% autophagic cells. The number of puncta was significantly lower in GC B cells (35% autophagic cells). The results could be validated by Western Blot analysis of LC3 revealing a shift from LC3I (unprocessed LC3) to LC3II (Figure 2D). These findings suggest basal autophagy activation under basal conditions in cHL. Interestingly, BL and DLBCL cells showed low LC3II levels under normal conditions. LC3II increased under starvation indicating basal autophagy activation by micro-environmental stress (Supplemental Figure 2).

To test LC3 processing in primary tissue we performed LC3II staining of cHL cases. 14 out of totally 17 cases were LC3II-positive (at least 50% positive tumor cells per case). Only 3 cases demonstrated negative staining (less than 50% positive tumor cells per case) comparable to that of the 3 cases tonsillar tissue and the 4 cases B-NHL tested (Figure 2E, Supplemental Table 5).

We found that CQ-treatment, which blocks lysosomal acidification, further increased LC3II in cHL cell lines suggesting intact autophagic flux (Supplemental Figure 3A). Consistently, treatment with the lysosome inhibitor pepstatin (Pep) dramatically increased LC3II under basal conditions (Supplemental Figure 4). Additionally, several studies have revealed that, besides LC3, p62 is preferentially degraded by autophagy [31, 32]. Thus, the total cellular expression levels of p62 inversely correlate with autophagic activity. We found indeed decreased p62 level in cHL cell lines, as compared to normal B cells. Tumor cells of cHL cases were mostly p62-negative (in 14/17 cases), the reactive infiltrate diffusely positive in some cases (Supplemental Figure 3B, Supplemental Table 5). In case of impaired autophagic flux, LC3II will not co-localize with EEA1, RAB7 and LAMP1. Thus, their distribution was determined by fluorescence microscopy (Supplemental Figure 3C). Indeed, LC3-II co-localized in L428 cells with EEA1, RAB7 and LAMP1. Moreover, after blockade of autophagosome degradation LC3II content increased in the autophagosomal fraction of L428 cell lysates, as expected in case of intact autophagic flux (Supplemental Figure 3D).

### Autophagy inhibition impairs cHL growth and affects mitochondrial metabolism

**Figure 3:**
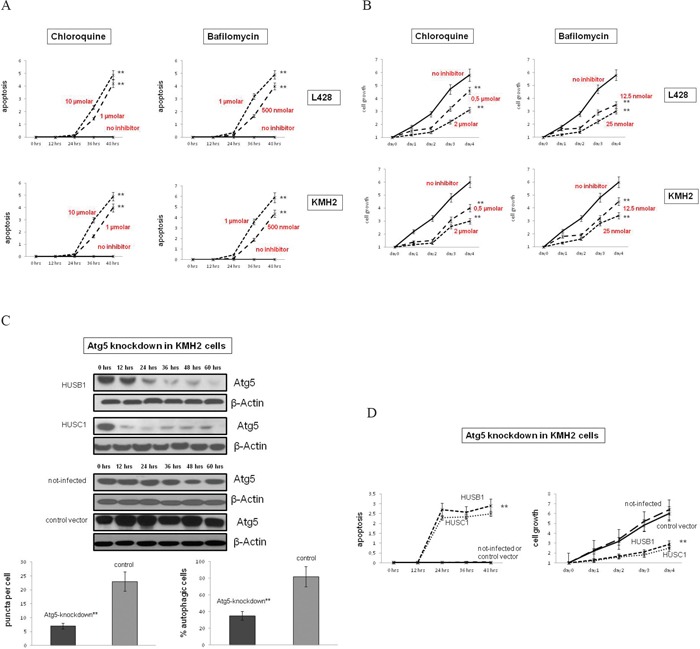
Autophagy inhibition reduces cell growth and induces apoptosis in cHL cells **A.** The cHL cell lines L428 and KMH2 were pre-incubated with the autophagy inhibitors CQ or Baf in two different concentrations of each. Apoptosis was determined by quantification of cytosolic nucleosomes over 48 hrs of incubation (ELISA). Difference in mean absorbance at A405 and A450 was measured. Results are expressed as means±SD for n=3 experiments for each cell line and each condition tested. ** Cell lines significantly different vs. control group (treated vs. untreated cells) by the t-test (p<0.001). **B.** Cell growth of cHL cell lines treated with CQ or Baf, as compared to untreated cells was determined by trypan blue exclusion method. Results are expressed as means±SD for n=3 experiments for each cell line and each condition tested. ** Cell lines significantly different vs. control group (treated vs. untreated cells) by the t-test (p<0.001). **C.** To inactivate autophagy genetically KMH2 cells were infected with two different lentiviral vectors encoding two different shRNAs (HUSB1, HUSC1) that inhibit ATG5-RNA expression. *Upper panel*, ATG5 expression in these cells and in control cells (infected with empty vectors or not infected) was determined by Western Blot analysis. β-Actin was used as a loading control. Shown is one representative Blot of n=3 experiments. *Lower panel*, To analyze the status-quo of autophagy knockdown cells and control cells were stained to LC3II and % autophagic cells was determined by confocal microscopy analysis. Results are expressed as means±SD for n=4 experiments. ** Cell lines significantly different vs. control group (knockdown vs. control cells (infected with empty vector or not-infected)) by the t-test (p<0.001). **D.** Apoptosis/cell growth of ATG5-knockdown and control KMH2 cells was analyzed by trypan blue exclusion method. ** Cell lines significantly different vs. control group (knockdown vs. control cells (infected with empty vector or not-infected)) by the t-test (p<0.01).

The prominent activation of basal autophagy in primary tissue and cell lines suggests that this process may be essential for growth and survival of cHL cells. In order to test this hypothesis, we treated cHL cell lines with CQ and bafilomycin (Baf) (Figure 3A, Supplemental Figure 5A, 5B). We found that both autophagy inhibitors induced apoptosis in L428 and KMH2 cells after 36 hrs of incubation, but had minimal effects on the BL cell line BL2, and the DLBCL cell line SUDHL6. Thus, in keeping with their basically elevated autophagy, cHL cells were sensitive to autophagy inhibition in contrast to the low autophagic BL2 and SUDHL6 cells. Consistently, we found that CQ and Baf markedly decreased proliferation of the two cHL cell lines L428 and KMH2 dependent upon the concentration of the inhibitors (Figure 3B). As the chemical inhibitors of autophagy used also affect lysosomal function, they may impact other cellular processes in addition to basal autophagy [29]. To inhibit autophagy more specifically we performed knockdown experiments using two different shRNAs to ATG5, which is as ubiquitin-like protein essential for autophagosome expansion and completion. Both shRNAs suppressed expression of the ATG5 protein and inhibited basal autophagy in KMH2 cells (Figure 3C). Additionally, KMH2 cells transduced with the lentiviral shRNAs exhibited increased rates of apoptosis and reduced cell growth, as compared to the control cells infected with lentivirus containing empty shRNA vector (Figure 3D). Both shRNAs used reduced expression of ATG5 in the B-NHL cell lines BL2 and SUDHL6, but had no significant effect on their cell viability and cell growth (Supplemental Figure 5C–5E). Taken together, these data illustrate that cHL cell lines depend on basal autophagy for continued cell growth and viability.

**Figure 4:**
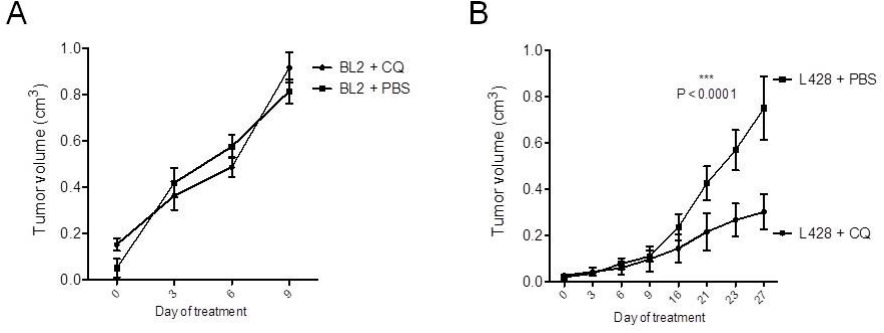
CQ-treatment significantly inhibits cHL growth *in-vivo* 1×10^5^ cells of BL cell line BL2 **A.** and 1×10^6^ cells of cHL cell line L428 **B.** were transplanted subcutaneously into the flanks of highly immunodeficient NSG mice (10 animals per cell line). After successful growth of xenograft tumors, recipient animals were divided into two cohorts for each cell line. Animals were either treated by daily intraperitoneal injections of 60 mg/kg CQ in PBS or with PBS only, as a control. Statistical analysis was performed by two-way ANOVA test. The statistical significance for the comparison of PBS and CQ-treated animals of the L428 group is indicated by the P-value shown in the graph.

As a next step, we wanted to investigate the potential therapeutic potency of CQ-induced inhibition of autophagy *in-vivo*. For this purpose, we subcutaneously transplanted NOD SCID γc^−/−^ (NSG) mice with the cHL cell line L428 or with the BL cell line BL2. After successful engraftment, xenograft-bearing mice were separated into two cohorts for each transplanted cell line and either treated by intraperitoneal injection of CQ or PBS, as a control. Reflecting the *in-vitro* results and as expected, CQ-treatment did not have any effect on the *in-vivo* growth of cell line BL2 (Figure 4A). However, sequential injections of CQ significantly impaired the growth of cHL cell line L428 (Figure 4B). Moreover, all treated animals tolerated well the applied CQ-dosage and daily injections did not cause any severe side effects.

To assess the role of autophagy in sustaining energy metabolism we analyzed the effect of CQ-inhibition on oxygen consumption in a panel of cHL cell lines. We observed severely decreased oxygen consumption upon CQ inhibition (Figure 6A). The ratios between the respiration rate after treatment with the uncoupler FCCP and ATP-synthase inhibitor oligomycin RCR_u/o_, indicative for the efficiency of oxidative phosphorylation (OXPHOS), were also reduced (Supplemental Figure 6A). Accordingly, glucose up-take and lactate production increased significantly upon CQ-treatment, while cellular ATP levels decreased (Supplemental Figure 6B). The reduction of OXPHOS upon CQ-treatment could reflect an accumulation of damaged mitochondria due to suppression of mitophagy. Indeed, in the presence of CQ expression of PINK-1 and Park2 and mitochondrial mass increased (Supplemental Figure 6C). As depolarized mitochondria are targeted for mitophagy, we additionally determined mitochondrial membrane potential upon CQ by FACS with the potentiometric dye TMRE. Mitochondria in cHL cells exhibited a significantly decreased membrane potential upon CQ-treatment (Supplemental Figure 6D). All parameters tested here were determined after 24 hrs CQ-treatment before cell viability changed to ensure that the effects seen in these experiments are not the consequence of apoptosis induction by CQ.

The decrease in OXPHOS upon CQ-treatment might be partially caused by the shift of glucose utilization towards lactate production that should lead to a shortage of carbon substrates for the TCA cycle [29]. We attempted to rescue the effect of CQ by supplementing the cells with excess pyruvate, which would bypass the substrate shortage. In fact, the addition of pyruvate partially mitigated the cytotoxic effect of CQ, as rates of apoptosis were lower upon CQ-inhibition after pyruvate-treatment. The treatment also partially rescued the decrease in cellular ATP (Supplemental Figure 6E, 6F).

## DISCUSSION

In summary, we showed that basically activated autophagy is a profound requirement for cHL pathology. Autophagy is constitutively activated in cHL cells, and is needed for continued malignant growth and energy conversion. This is a result that has never been reported before. We propose that this dependence on basal autophagy is exploitable for therapeutic benefits.

### Autophagy is constitutively activated in cHL in cHL cell lines and cHL patients

Although lymphoma cell lines were grown under conditions with optimal substrate supply, the expression analysis showed the same pronounced up-regulation of key autophagy proteins in cHL cell lines as in patients' primary tissue (Figure 1A, 1B, Supplemental Figure 1A, Supplemental Table 4). This indicates that the changes in autophagy observed *in-vivo* in patients is reflected *in-vitro* in cHL cell lines. The most important autophagy-relevant protein is LC3 [15–20, 29]. Increased levels of total LC3 have been linked in solid tumors with activated autophagy accompanied by over-expression of other autophagy-relevant proteins [29]. However, it is still controversially discussed whether the respective elevated protein levels unambiguously indicate activated autophagy, since the relationship between gene expression levels and autophagy *per se* is uncertain [29]. Thus, we tested LC3 processing. LC3II is specifically localized to autophagosomes that accumulates in cells upon autophagy activation [15, 16, 29]. We found LC3II over-expressed in cHL cell lines and most tumor cells of primary cases clearly suggesting activated autophagy *in-vitro* and in cHL patients (Figure 1, Figure 2, Supplemental Figure 2). Collaborating with these results, numbers of autophagosomes were high in cHL cells, and furthermore, high p62 levels and increased autophagosome numbers upon CQ treatment indicated intact autophagic flux (Figure 2, Supplemental Figure 3, Supplemental Figure 4). This suggests basal autophagy activation as a cell-autonomous mechanism that is not induced by nutrient deprivation like in highly aggressive tumors [35], or by other environmental stressors [36]. Otherwise basal autophagy would not be increased using cHL cell lines in a model that did not mimic the tumor microenvironment.

The basal autophagy activation is probably a characteristic feature of cHL cells. Low expression levels of autophagy markers and decreased LC3 processing indicated autophagy repression under basal conditions in BL and DLBCL cell lines (Supplemental Figures 1B, 1C, 1E, 2). Moreover, low basal autophagy level was also found in T-NHL cells, as characterized by Mitou and Frentzel et al. in a recent study about the role of autophagy in ALCL [37]. Nutrient-deprived growth of BL2 and SUDHL6 cells induced autophagy suggesting that these cells carry no defects leading to autophagy repression, but up-regulate it upon environmental stressors. Consistently, malarial prophylaxis with CQ decreases the incidence of BL indicating that autophagy is maybe important in BL development in patients, where the presence of the host microenvironment induces alterations in nutrient supply [38]. In contrast to BL and DLBCL, cHL cells need autophagy for cell maintenance even under optimal nutrient supply. Other studies demonstrated increased basal autophagy that was important for cell survival in follicular lymphoma or multiple myeloma cells as well [39, 40]. We propose that the basal autophagy activation in cHL could in part be explained by the genetic instability, which is a characteristic feature of cHL cells [41]. High genetic instability leads to a high degree of protein misfolding [20, 42]. Autophagy inhibition could thus cause accumulation of mis-folded proteins, which could entail tumor cells' demise [20]. Consistently, we found basically increased Park2 mediating ubiquitination and proteolytic destruction of terminally mis-folded proteins [20].

### Autophagy could probably be a good target in cHL therapy

The dependence of cHL on basal autophagy may provide a much needed target for therapeutic intervention and a starting point to develop new strategies in cHL treatment. Autophagy modulation has been shown to be a good potential therapeutic target in diverse diseases [43, 44]. The results of our functional experiments suggest that basal autophagy promotes cell survival and proliferation in cHL and thus CQ might be suitable for cHL treatment. Knockdown of ATG5 and CQ-treatment increased rates of apoptosis in cHL cell lines and inhibited cell growth in cell culture experiments and *in-vivo* in mice (Figure 3). Autophagy may act to promote tumor development in other types of lymphomas, and thus autophagy inhibition is discussed as a novel therapeutic strategy in other lymphoma entities than cHL [45]. For example, in a T-cell lymphoma mouse model, CQ-treatment impaired tumor formation and enhanced animal survival [46]. If this work in particular provides preclinical evidence that CQ could be effective in the prevention of T-cell lymphoma, other studies suggest that inhibition of autophagy could be important in the treatment of B-cell lymphomas [47, 48]. Finally, all these studies support the hypothesis that autophagy correlates with tumor progression. On the other hand, contradicting other reports suggest that inactivation of autophagy supports tumorigenesis in lymphoma reflecting the view of its role as a tumor suppressive mechanism [49].

The positive role of autophagy in cHL cell maintenance is also consistent with emerging reports about solid tumors exhibiting activated autophagy with the function of tumor promotion [29, 50]. However, our results do not preclude a tumor suppressing function during tumorigenesis in cHL. It is nevertheless possible that the role of basically activated autophagy is biphasic in cHL, as it has been suggested for other tumor types [29]. During tumorigenesis autophagy suppression might trigger the pro-tumorigenic genomic instability essential for tumor formation, whereas in highly metabolically active, established tumor cells promote tumor growth.

### Autophagy promotes continued energy conversion in cHL cells

OXPHOS, the mitochondrial membrane potential and the expression of the mitophagy markers PINK-1 and Park2 decreased upon autophagy inhibition in cHL cells (Supplemental Figure 6). Both, PINK-1 and Park2, which have been linked to increased mitochondrial turnover associated with increased OXPHOS activity, were basically over-expressed in cHL cells suggesting high mitophagy levels in addition to strong autophagy in cHL. Mitophagy has been shown to be important in mitochondrial quality control by degrading damaged organelles to maintain a stable cellular pool of excellent working organelles [51, 52]. It seems to be pivotal in cHL for two reasons, in sustaining OXPHOS by providing metabolic intermediates on one and mitochondria of high quality on the other hand. We thus propose that elevated mitophagy represents an important aspect in cHL biology.

## MATERIALS AND METHODS

### Cell culture and patient samples

Lymph node samples from 17 patients with cHL and 3 patients with tonsillitis were collected from the Dr. Senckenberg Institute of Pathology, University Hospital of Frankfurt am Main, Germany. To obtain GC B cells, CD77^+^ cells were isolated from tonsillar cell suspension received from patients who underwent routine tonsillectomy. The cell suspension was pre-incubated with the mouse anti-CD77 antibody (mouse IgM antibody from Abcam) and then treated with anti-mouse IgM microbeads before subjecting to magnetic cell sorting. B cells were cultured in RPMI Glutamax with 20% of FBS and addition of rabbit anti-IgM (15 μg/ml from Sigma-Aldrich). All cell lines were obtained from the *Deutsche Sammlung von Mikroorganismen und Zellkulturen (DSMZ)* and cultured with addition of penicillin/streptomycin in RPMI Glutamax with 10% of FBS (the cHL cell lines L428, L1236, KMH2, L540, HDLM2; the BL (Burkitt lymphoma) cell lines BL2, BL41; the DLBCL (Diffuse large B cell lymphoma) cell lines OCI-Ly19, SUDHL6), in 80% Iscove's MDM with RPMI 1640 (at 4:1) and 20% FBS (the cHL cell line UHO1), in 90% RPMI 1640 with 10% FBS and 10 μg/ml human insulin (MCF7 cells) or in 90% Dulbecco's MEM with 5% FBS and 5% horse serum (8988T cells). All cell lines used in this study have been authenticated by STR profiling in 2015 by our group.

### Respiration measurements

Oxygen consumption was determined by high-resolution respirometry [25]. After recording the basal respiration, 1 μg/ml oligomycin was added to inhibit ATP synthesis and subsequently the uncoupler FCCP was applied to determine respiratory capacity. The FCCP concentration required for maximal stimulation had to be adapted to the different cHL cell lines: 2 μM (L428, KMH2) and 3 μM (L1236).

### Fluorescence microscopy

Cells were plated on Poly-L-lysine coated cover slips, fixed with 4% para-formaldehyde, permeabilized with TritonX100 and blocked with 5% BSA, before subjecting to the primary and the secondary antibody (summarized in Supplemental Table 1). Counterstaining was performed with DAPI (Invitrogen) followed by mounting the cover slips on glass slides. Observation was performed with the Olympus FluoView FV1000 confocal laser scanning microscope.

### FACS analysis

The CytoID Green autophagy dye (Enzo Life Science), and the potentiometric dye TMRE (Abcam) were used according to the manufacturers' instructions. The mean fluorescence of a total of 1*10^4^ cells per cell type tested was determined by flow cytometry and normalized on the protein content.

### Immunostainings and Western blots

Immunohistochemistry of paraffin-embedded tissue, cell lysates, SDS-PAGE and Western blotting were performed according to standard protocols [26–28]. Antibodies and experimental conditions are summarized in Supplemental Table 2 and 3. For detection of oxidized proteins the oxyblot protein oxidation detection Kit (Millipore) was used according to the manufacturers' instructions.

### Functional *in-vitro* studies

Cells were grown in 24-well plates at counts of 5*10^5^/ml and pre-incubated with chloroquine, bafilomycin, and pepstatin (purchased from Sigma-Aldrich). Changes in cell death were analyzed using the Cell Death Detection ELISA Kit from Roche. Cellular ATP levels were determined with the ATP Colorimetric Assay (Biovision) before cell viability changed (at a cell viability of at least 90%). For cell growth analysis live cells were counted daily by trypan blue exclusion method and for each inhibitor used the dose was determined that did not affect cell viability over a time period of 96 hrs (chloroquine (0.5 or 2 μM), and bafilomycin (12.5 or 25 nM). For metabolic studies lactate levels were measured by the lactic acid assay kit (Abcam), glucose consumption using the glucose quantification assay (BioVision). Pyruvate (Sigma-Aldrich) was added to the cell culture medium at 50 μg/ml and the anti-oxidant N-acetyl cysteine (Sigma-Aldrich) was used at a concentration of 5 μg/ml.

Lentiviral shRNA (Origene technologies, Rockville, USA) targeted to autophagy-related protein-5 (ATG5) RNA was used to knockdown ATG5 protein expression. Two separate ATG5 knockdown cell lines were generated for each cell line using two different lentiviral vectors encoding for different shRNA sequences (HuSH-B1 and HuSH-C1 from Origene). Briefly, lentivirus was produced by transfecting 293T cells with 7.5 μg/ml transfer plasmid, 12.5 ug/ml M334 vector (packaging plasmid) and 1 μg/ml M5 vector (envelop plasmid). 48 hrs after 293T cell transfection, supernatant containing lentivirus was collected and immediately used for infection or stored at −80°C. For infection, 2 ml of supernatant containing lentivirus was added to each well of a 6-well plate containing 1×10^5^ cells. Cells were incubated with lentivirus for 48 hrs and next transferred to a 75 mm flask. Assessment of ATG5 protein knockdown was determined when cells were approximately 70% confluent. Hereafter, infected and not-infected cell lines were cultured under normal cell culture conditions. Apoptosis of a total of 1*10^6^ cells was analyzed using the Roche cell death ELISA each 12 hrs starting at confluence for a total time period of 48 hrs. Control cells were infected with lentivirus containing empty shRNA vector or not infected.

### CQ *in-vivo* treatment studies

NOD SCID γc^−/−^ (NSG) mice (The Jackson Laboratory) were used for CQ *in vivo* treatment studies. Mouse experiments were performed in accordance with the local animal experimentation guidelines and approved by the regional council (Regierungspräsidium, Darmstadt, Germany, protocol number: F21/04). Mice were kept and bred according to the guidelines of the Federation of European Laboratory Animal Science Associations (FELASA) in the animal facility of the Georg-Speyer-Haus (Frankfurt am Main, Germany).

For xenografts, 1×10^6^ L428 or 1×10^5^ BL2 cells in a total volume of 100 μl Hanks buffered saline solution (PBS) were injected subcutaneously into the lower right flank of recipient mice (n=10 per cell line) using an insulin syringe (BD). After tumor engraftment, for each cell line, mice were separated into two groups. Animals of the CQ group daily received an intraperitoneal injection with CQ at 60 mg/kg in 100 μl PBS, whereas control animals were injected daily with 100 μl PBS only. Tumor size was determined every 3 days using calipers and tumor volumes were calculated (length x width^2^/2). General health status of all animals was monitored daily. The experiment was followed up until tumors reached a volume of 0.8 cm^3^.

## SUPPLEMENTARY FIGURES AND TABLES




