# Supplementary material for: Basal autophagy is pivotal for Hodgkin and Reed-Sternberg cells' survival and growth revealing a new strategy for Hodgkin lymphoma treatment
Source: Oncotarget. 2016 Jun 27;7(29):46579–88. doi: 10.18632/oncotarget.10300 (PMC5216819; doi:10.18632/oncotarget.10300)
Supplement: Supplementary file 1 [file oncotarget-07-46579-s001.pdf]

Basal autophagy is pivotal for Hodgkin and Reed-Sternberg cells' survival and growth revealing a new strategy for Hodgkin lymphoma treatment

SUPPLEMENTARY FIGURES AND TABLES

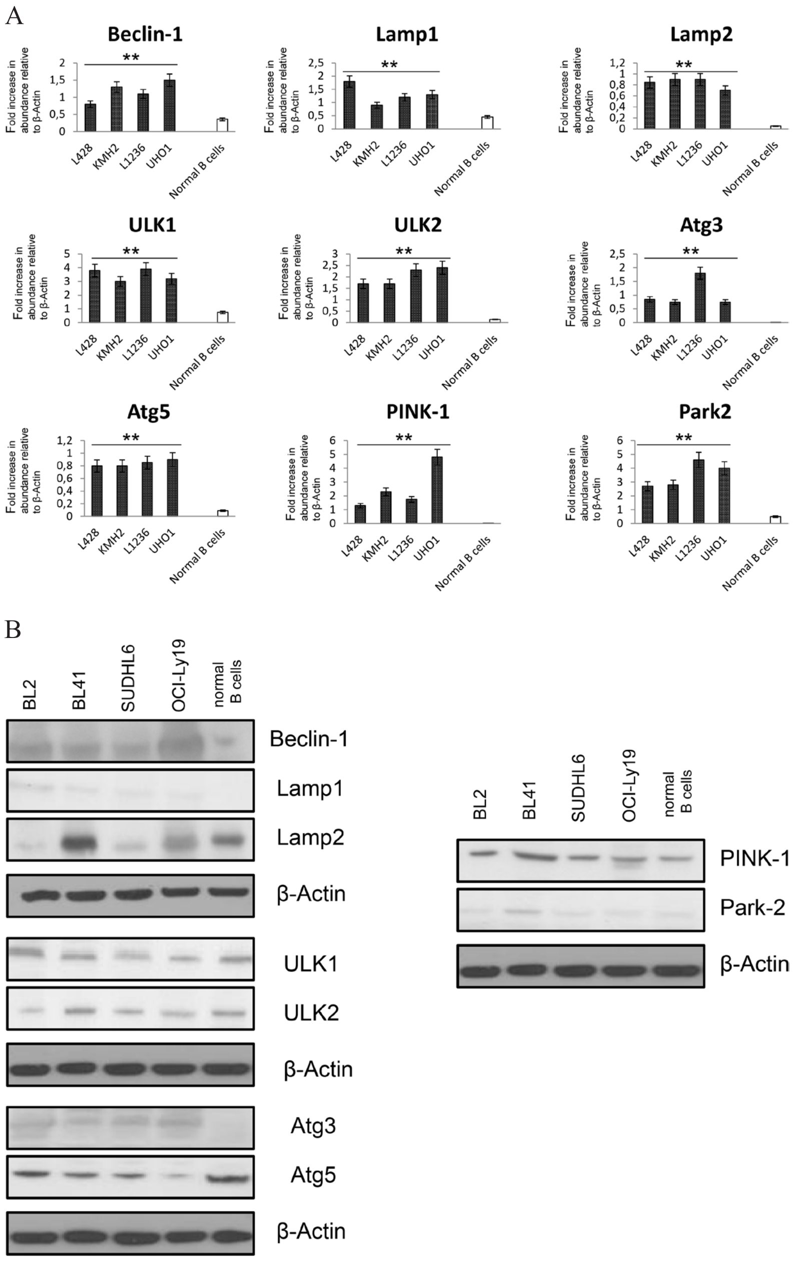

Supplementary Figure S1: A. Densitometric quantification of autophagy-related proteins in cHL cell lines. Band intensities were analyzed using ImageJ and the relative intensity was normalized for each band on β-Actin expression. Data are expressed as means±SD for n=5 experiments. ** p<0.001 cHL cell lines vs. normal B cells. **B.** Autophagy-related proteins are weakly expressed in other B-cell lymphomas. Expression of autophagy-related proteins were determined by Western Blot in BL (BL2, BL41), DLBCL cell lines (SUDHL6, OCI-Ly19), and normal B cells. Shown is one representative Blot for each protein tested of a total of n=3 experiments. β-Actin was used as a loading control.

(*Continued*)

C

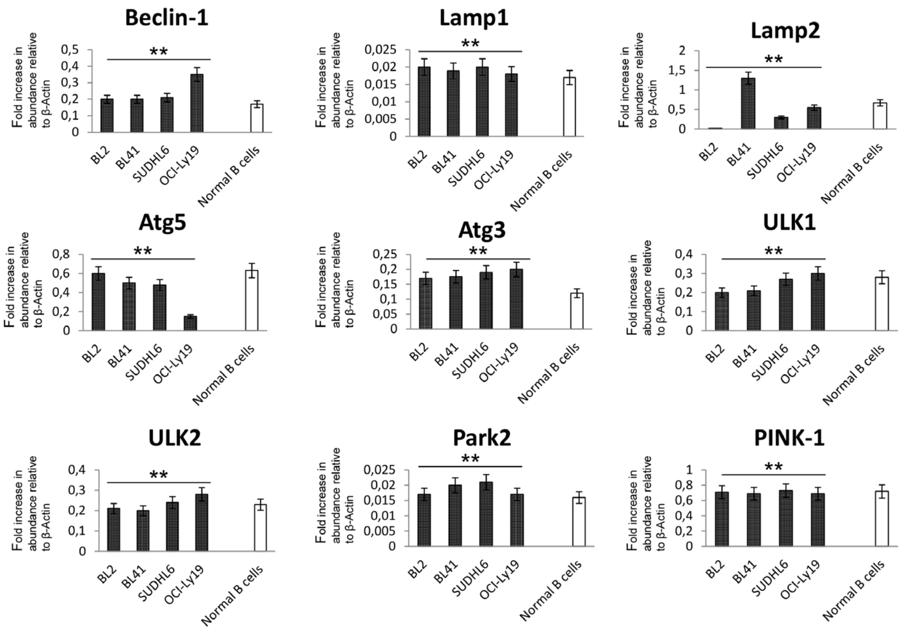

D

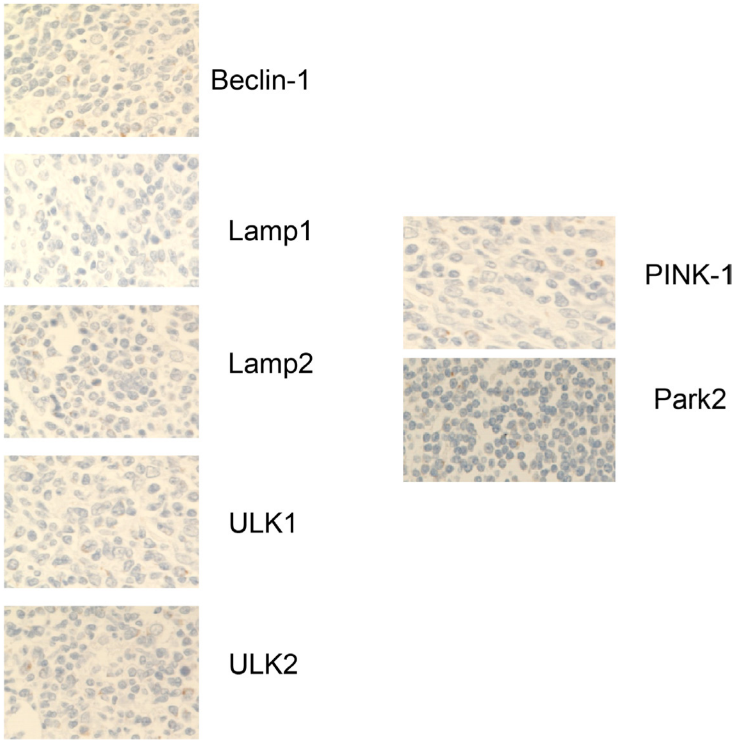

Supplementary Figure S1 (*Continued*): C. Densitometric quantification of autophagy-related proteins in BL and DLBCL cell lines. Western Blot analysis of autophagy-related proteins in the BL cell lines BL2 and BL41 and the DLBCL cell lines SUDHL6 and OCI-Ly19. Band intensities were analyzed using ImageJ and the relative intensity was normalized for each band on β-Actin expression. Data are expressed as means±SD for n=3experiments. **p<0.001 NHL cell lines vs. normal B cells. **D.** Autophagy-related proteins are weakly expressed in non-malignant tonsillar tissue. Immunohistochemical stainings of paraffin sections of lymph nodes from healthy donors were performed for autophagy proteins. Pictures of tonsills were taken at 400x magnification.

(*Continued*)

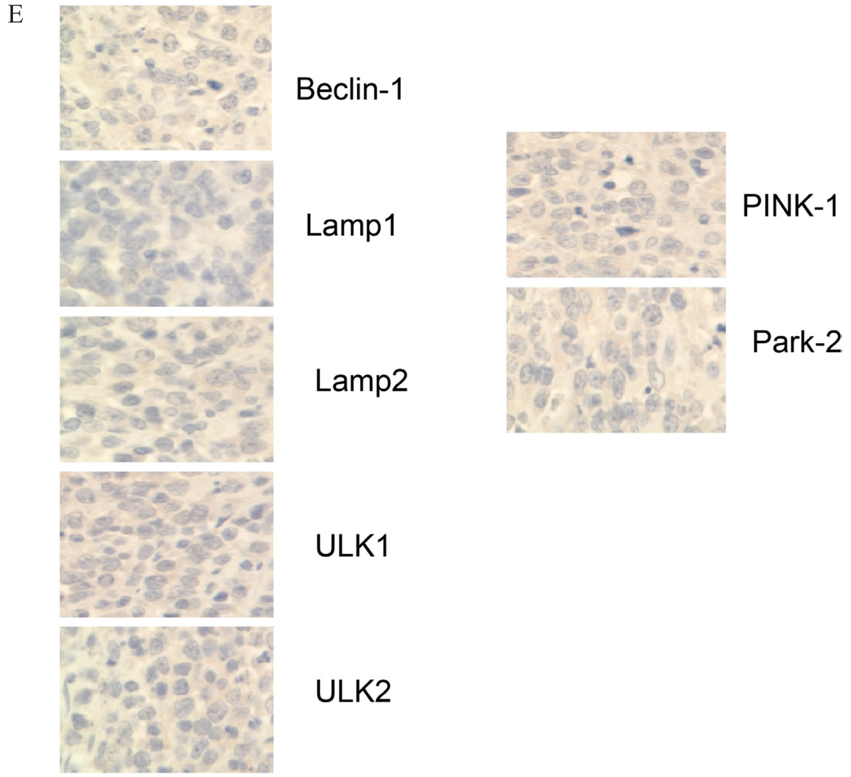

Supplementary Figure S1 (*Continued*): E. Autophagy-related proteins are weakly expressed in other GC B-cell lymphomas. Immunohistochemical stainings of paraffin sections of lymph nodes from patients with BL were performed for autophagy proteins. Totally 4 cases were analyzed. Pictures of cases were taken at 400x magnification.

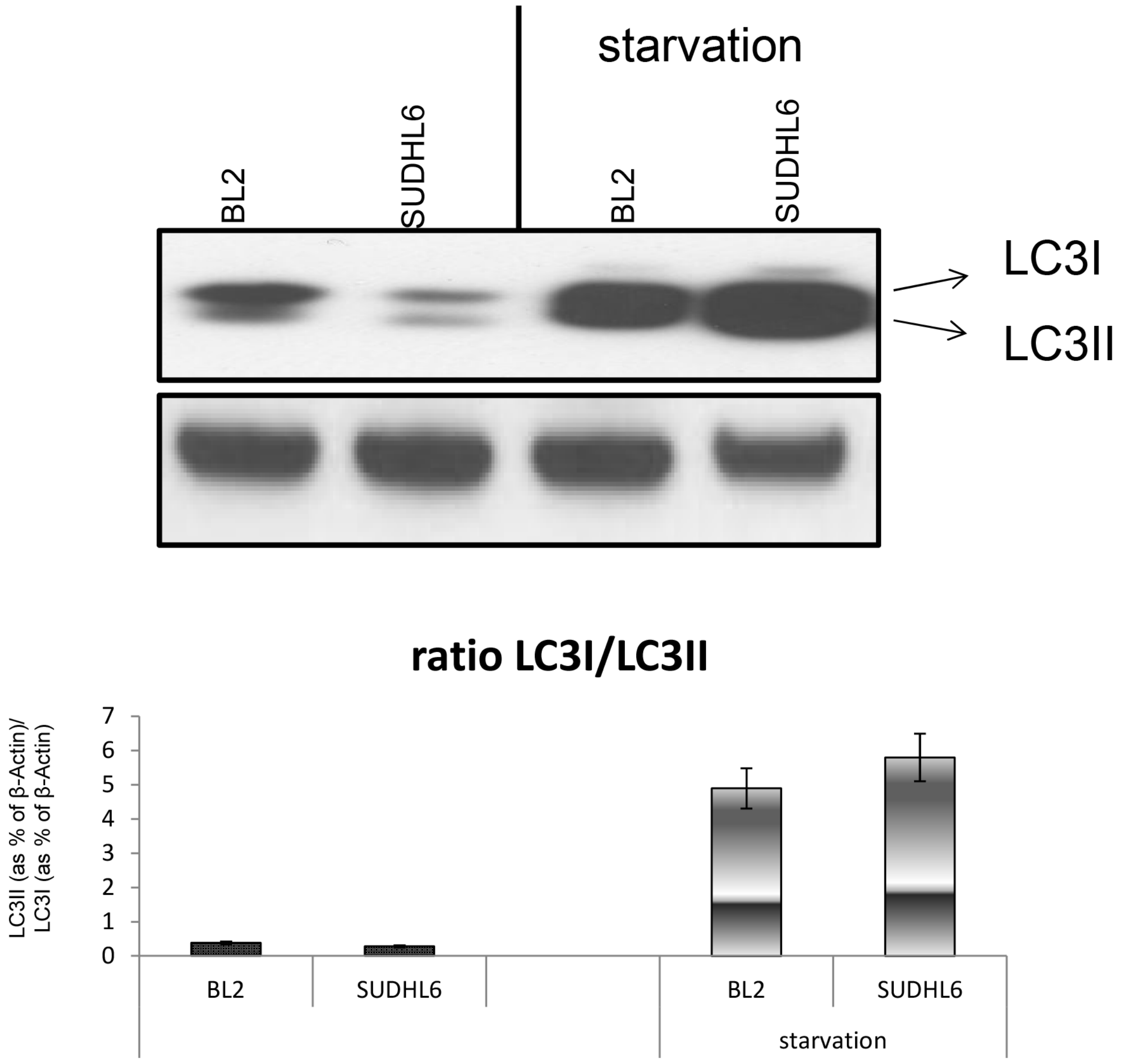

Supplementary Figure S2: LC3 processing is low in other B-cell lymphomas. Cells were grown under normal cell culture conditions and lysates were prepared from cells that were no longer left in culture than 48 hrs without passaging to prevent autophagy-inducing nutrient deprivation (left panel). To investigate whether autophagy is induced upon nutrient stress cell lines were cultivated in growth medium containing 5% FBS imstead of 10% (right panel). Shown is one representative Western Blot of a total of n=3 experiments for each analysis (lower panel). Densitometric analysis was performed using ImageJ. Results are expressed as means±SED.

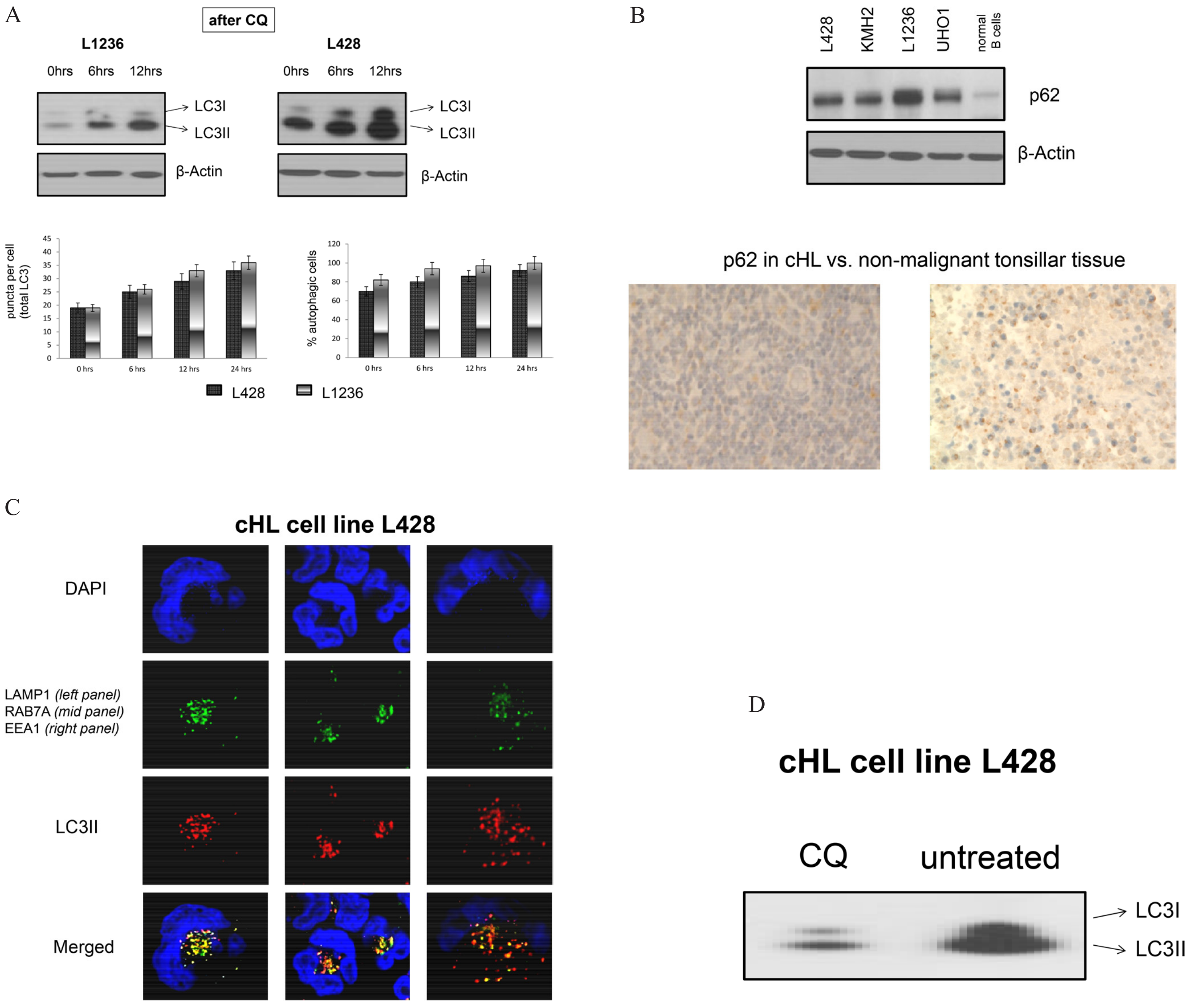

Supplementary Figure S3: Autophagic flux is intact in cHL cells. A. Western Blot analysis of LC3 expression over a time period of 12 hrs in the cHL cell lines L1236 and L428 (*upper left and right panel*), analysis of LC3II puncta per cell (*lower left panel*), and % of autophagic cells (*lower right panel*) were performed in CQ-treated cHL cells. **B.** p62 expression was analyzed by Western Blot in cHL cell lines and normal B cells (*upper panel*) and by immunohistochemistry in cHL cases (*lower panel*). **C.** Expression of LC3II, LAMP1, EEA1, and RAB7A in the cHL cell line L428 was determined by immunofluorescence microscopy. **D.** LC3 was analyzed in the autophagosomal fraction from L428 cells, which were CQ-treated or untreated. A, B, D for each experiment one representative Blot is shown of totally n=3 experiments.

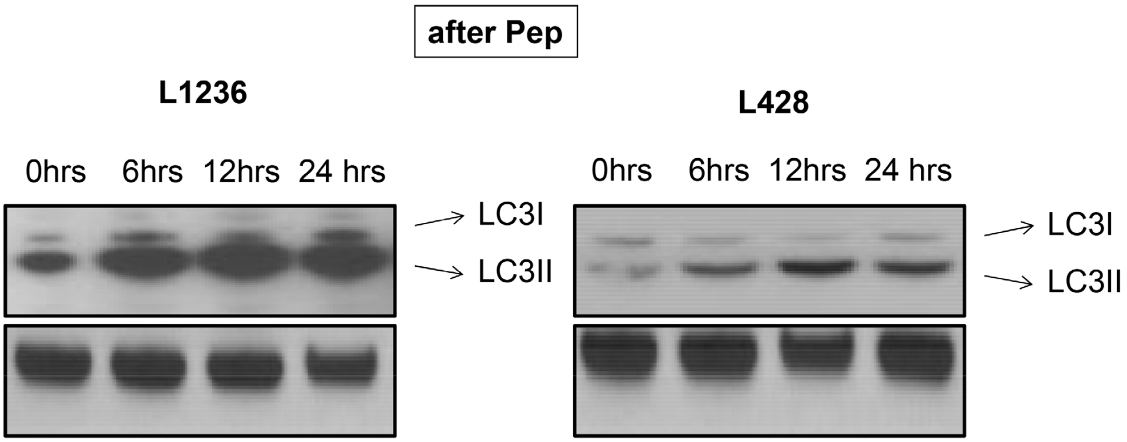

Supplementary Figure S4: Autophagic flux is intact in cHL. Western Blot analysis of LC3 expression of Pep-treated cells over a time period of 24 hrs. Shown is one representative Blot of a total of n=3 experiments. Pepstatin was used at a concentration of 1 µmolar in these experiments.

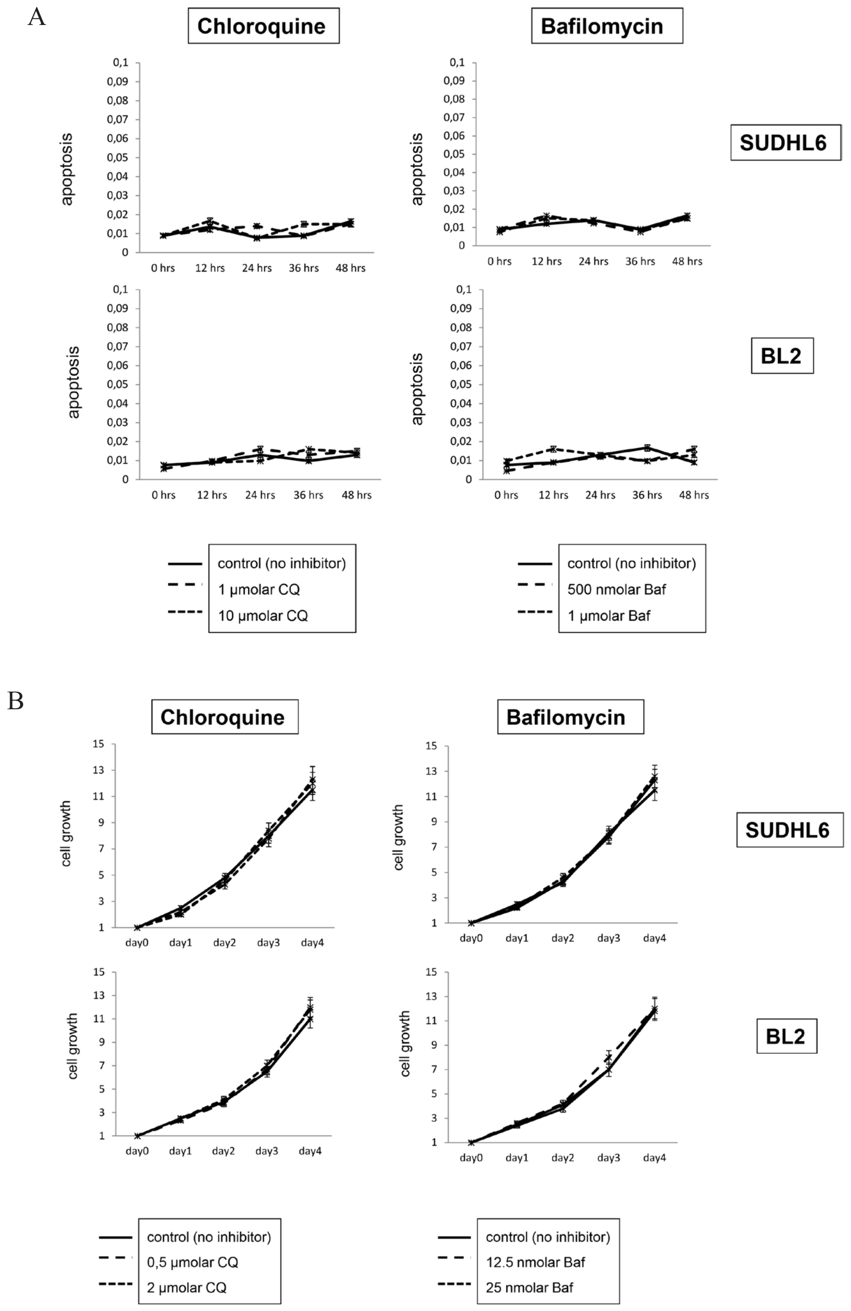

Supplementary Figure S5: Autophagy is not pivotal for B-NHL. A. The DLBCL cell lines SUDHL6 and the BL cell line BL2 were pre-incubated with the autophagy inhibitors CQ or Baf in two different concentrations of each. Apoptosis of lymphoma cells was determined by quantification of cytosolic nucleosomes over 48 hrs of incubation (ELISA). Difference in mean absorbance at A405 and A450 was measured. Results are expressed as means±SD for n=3 experiments for each cell line and each condition tested. **B.** Cell growth of lymphoma cell lines treated with CQ or Baf, as compared to untreated cells was determined by trypan blue exclusion method. Results are expressed as means±SD for n=3 experiments for each cell line and each condition tested.

(*Continued*)

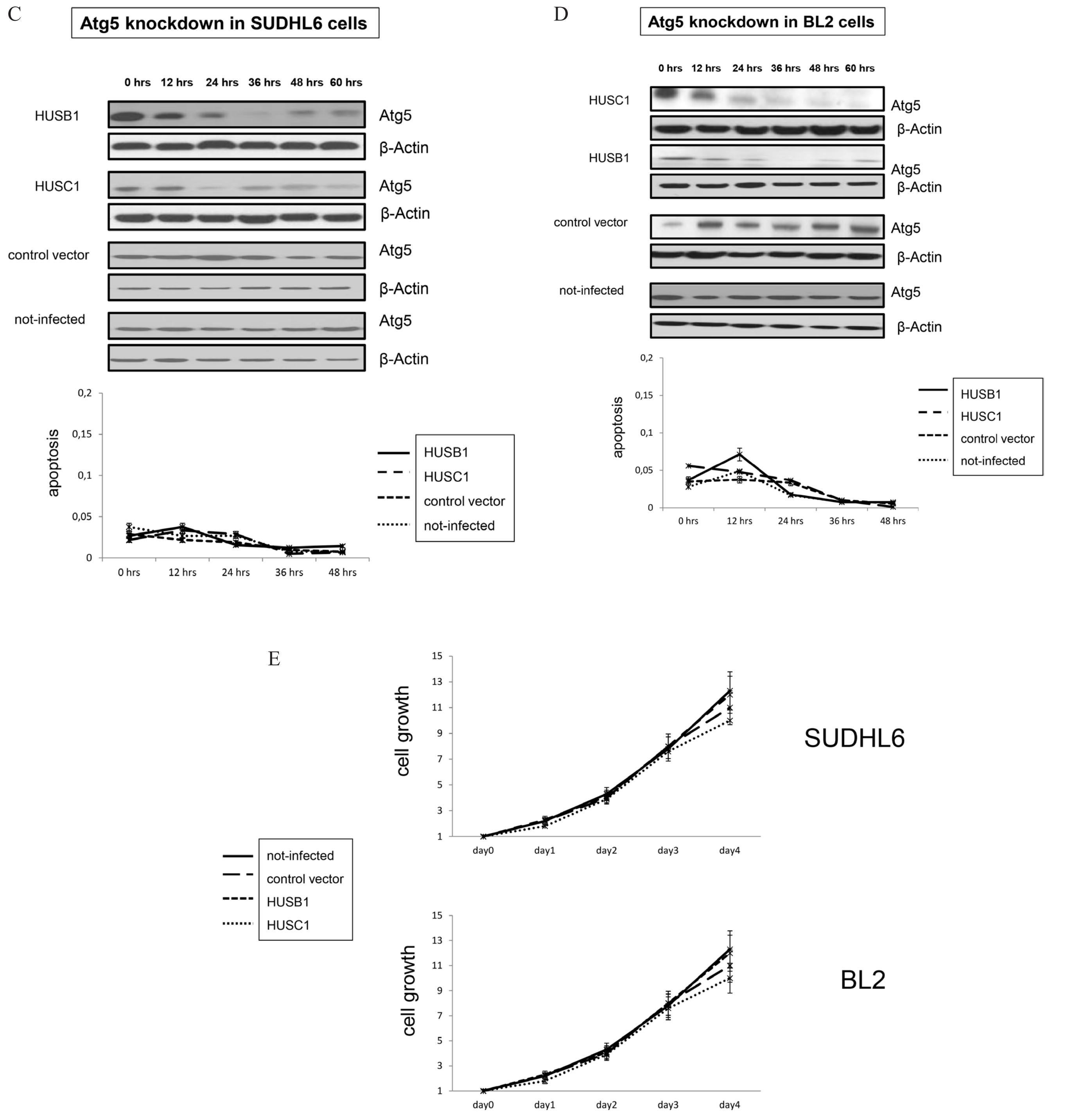

Supplementary Figure S5 (*Continued*): C-D. To genetically inactivate autophagy in B-NHL SUDHL6 and BL2 cells were infected with HUSB1, HUSC1 that inhibit ATG5-RNA expression. *Upper panel*, ATG5 expression in knockdown- SUDHL6 or -BL2 cells and in control cells (infected with empty vector (control vector) or not-infected) was determined by Western Blot analysis. β-Actin was used as a loading control. Shown is one representative Blot of n=3 experiments. *Lower panel*, apoptosis of ATG5-knockdown and control SUDHL6 or BL2 cells was analyzed. **E.** Cell growth of ATG5-knockdown and control SUDHL6 or BL2 cells was analyzed.

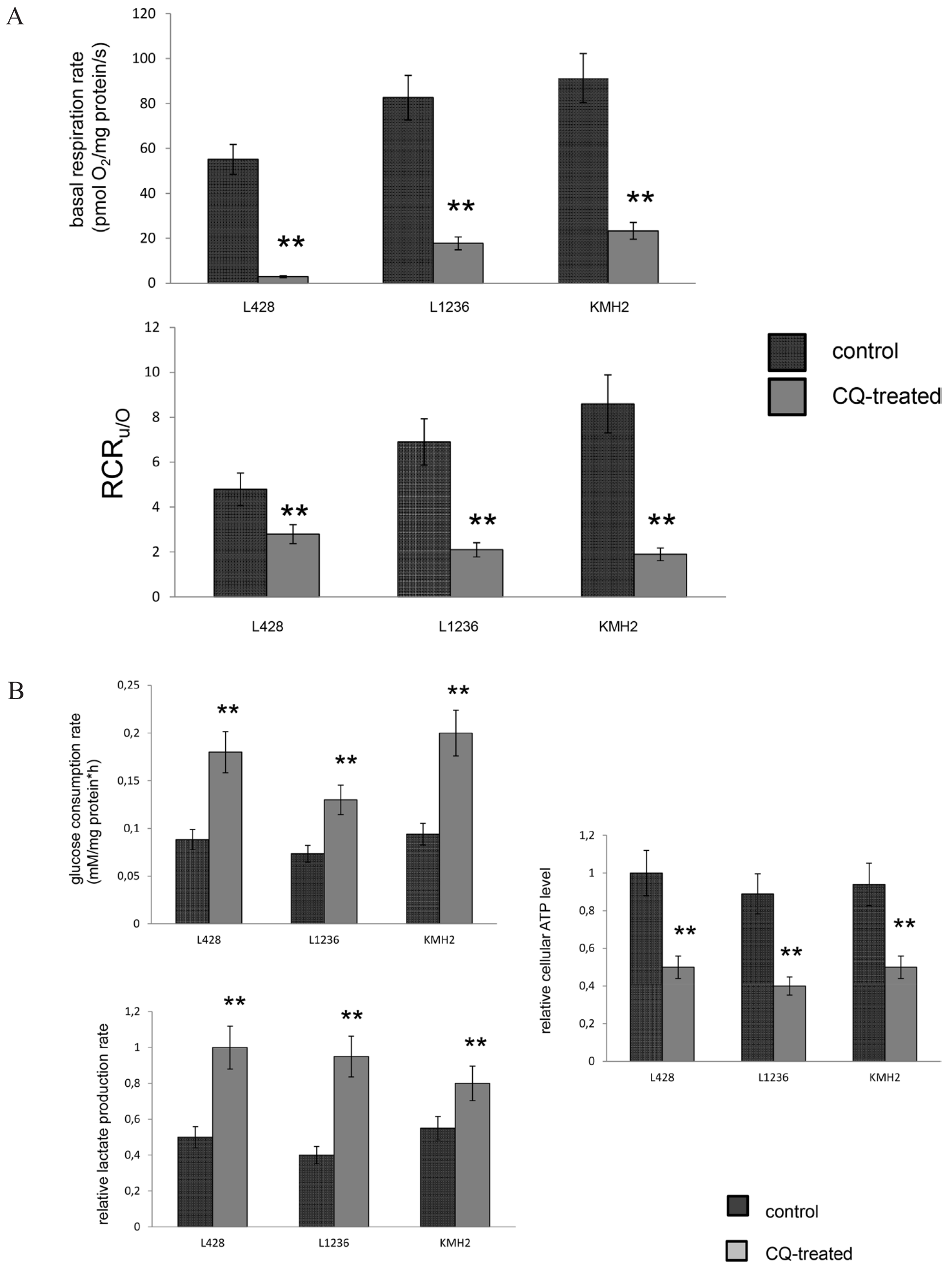

Supplementary Figure S6: cHL cells require autophagy for continued mitochondrial function. A. Oxygen consumption rates in pmol/mg protein/s (*upper panel*) and the ratio between the respiration rate after treatment with the uncoupler FCCP and the respiration rate following inhibition of ATP-synthesis by oligomycin $RCR_{u/o}$ (*lower panel*) were measured by high-resolution respirometry (Oxygraph-2k) in CQ-treated and untreated cHL cells. **B.** (*upper left panel*), Glucose consumption was measured by photometric quantification of glucose in cell culture supernatants by the glucose quantification assay after 6, 12 and 24 hrs of cell culture and glucose consumption rates were calculated in mM/mg protein*h. B (*lower left panel*), CQ-treated and untreated cHL cells were cultured for a total time period of 24 hrs and supernatants were collected for photometric quantification of lactic acid production after 6, 12 and 24 hrs of cell culture. Lactate levels were measured as [(mg lactic acid/L)/mg protein]. Lactate production rate was calculated for all tested cell types and each condition in [(mg lactic acid/L)/mg protein*h] and the highest rate was set to 1. B (*right panel*), Cellular ATP levels were determined after 24 hrs of cell culture in CQ-treated and untreated cHL cell lines setting the highest value to 1.

C

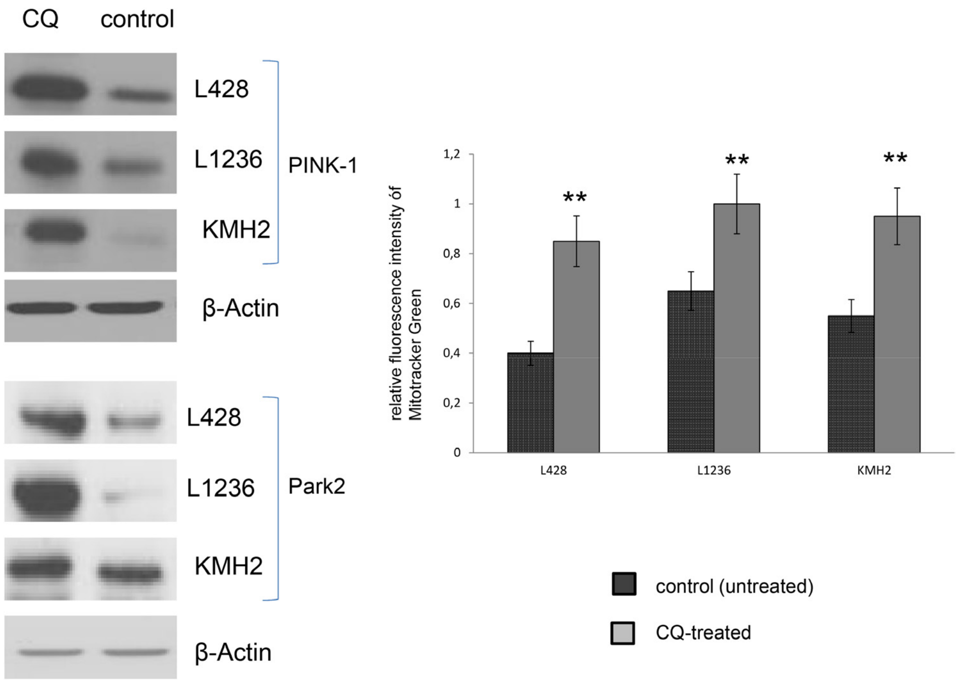

D

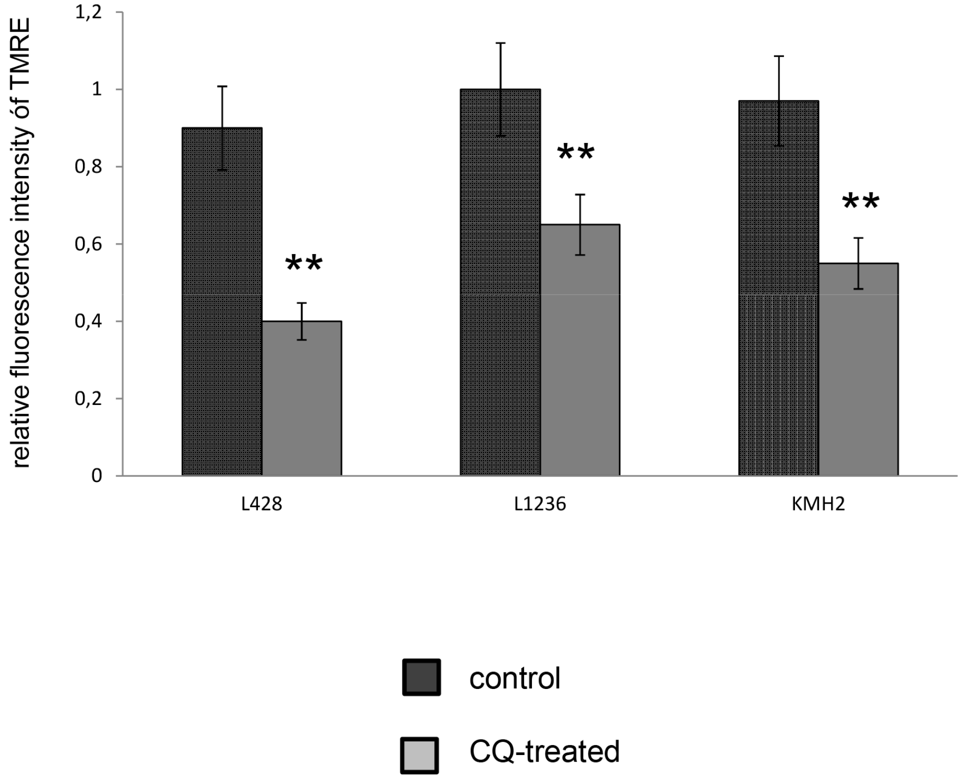

Supplementary Figure S6 (*Continued*): C. (*left panel*), The mitophagy markers PINK-1 and Park2 were determined in CQ-treated and untreated cHL cell lines after 24 hrs of cell culture. C (*right panel*), To determine the mitochondrial mass CQ-treated and untreated cell lines were stained with Mitotracker Green, the up-take of which is not dependent on the mitochondrial membrane potential. Mean fluorescence was analyzed by FACS setting the highest value to 1. **D.** To investigate mitochondrial membrane potential CQ-treated vs. untreated cHL cells were pre-incubated with TMRE. Mean fluorescence was analyzed by FACS setting the highest value to 1.

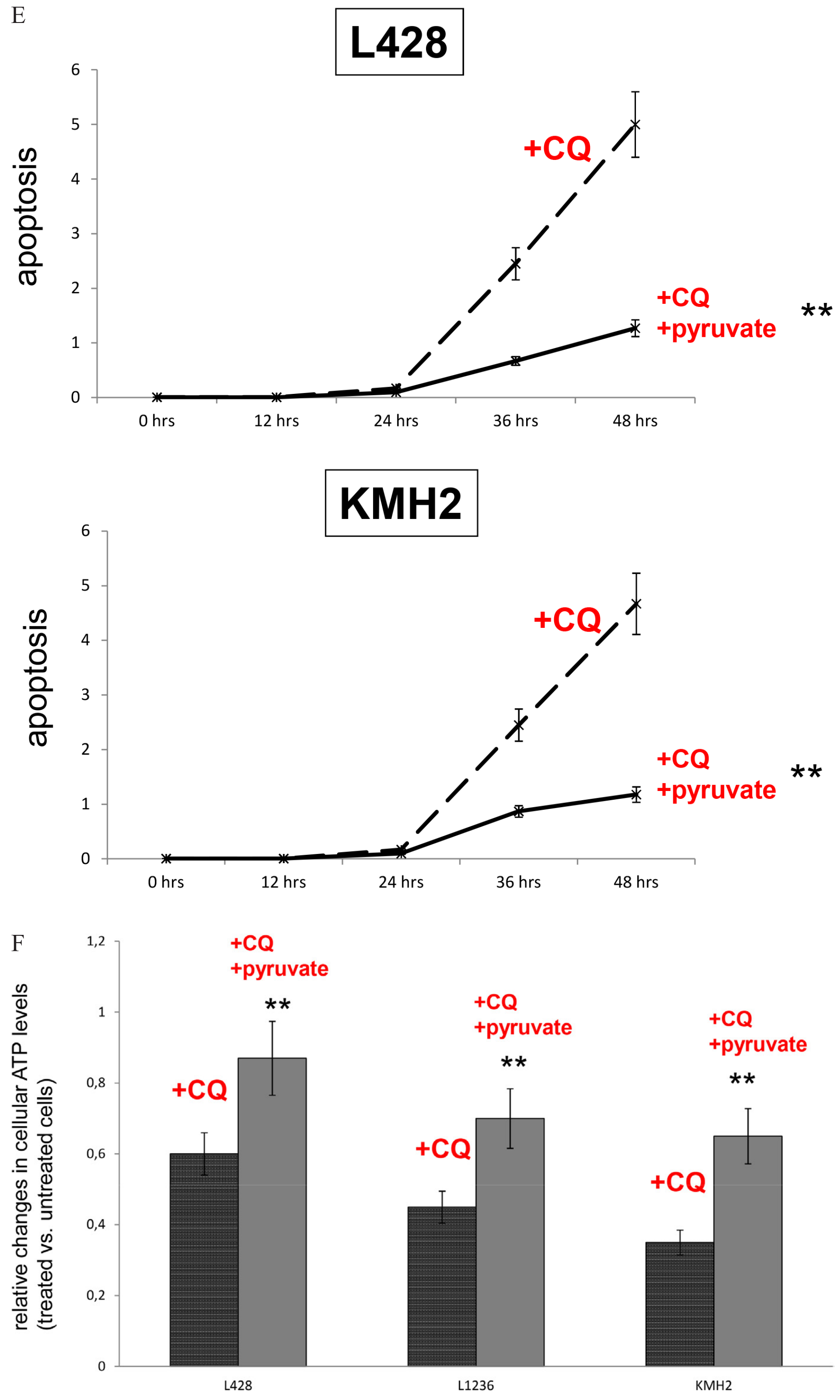

Supplementary Figure S6 (*Continued*): E. Apoptosis was determined in CQ-treated cHL cells with or without addition of pyruvate. **F.** Relative changes in cellular ATP levels in CQ-treated cells with pyruvate added vs. without pyruvate were determined after 24 hrs of cell culture. A-D, ** Lymphoma cell lines significantly different from control group by the t-test (treated vs. untreated). E, F, ** Lymphoma cell lines significantly different from control group by the t-test (with pyruvate vs. without pyruvate).

Supplementary Table S1: Summary of antibodies used for fluorescence stainings

Protein	Antibody	Dilution
LC3II	AP1805a, Abgent	1:100
EEA1	ab70521, Abcam	1:500
RAB7A	sc-271608, Santa Cruz Biotechnology	1:100

Supplementary Table S2: Summary of antibodies used for Western Blot analysis

Protein	Antibody	Dilution
Beclin-1	NBP1-00084, Novus Biologicals	1:1000
Lamp1	NBP1-43364, Novus Biologicals	1:2000
Lamp2	NBP1-71692, Novus Biologicals	1:1000
ULK1	#5723-1, Epitomics	1:5000
ULK2	#HPA009027, Atlas antibodies	1:1000
ATG3/ATG5	Autophagy antibody sampler Kit, Cell Signaling	1:1000
PINK-1	LOM-B3384-50, Labomics	1:10000
Park2	ab53042, Abcam	1:1000

Supplementary Table S3: Summary of antibodies used for immunohistochemistry

Protein	Antibody	Dilution
Beclin-1	NBP1-00084, Novus Biologicals	1:100
Lamp1	NBP1-43364, Novus Biologicals	1:250
Lamp2	NBP1-71692, Novus Biologicals	1:100
ULK1	#5723-1, Epitomics	1:50
ULK2	#HPA009027, Atlas antibodies	1:100
LC3II	AP1805a, Abgent	1:50
p62	PW9860, Enzo Life Science	1:500

Supplementary Table S4: Key autophagy markers are up-regulated in cHL primary tissue. Immunohistochemistry score of tumor cells and reactive infiltrate as percentage of cHL cases and control tissue (normal non-malignant lymphoid tissue). Score 0 = negative staining (<50% cells), score 1 = positive (>50% cells). The stainings were evaluated independently by two pathologists without knowledge of other findings.

See Supplementary File 1

Supplementary Table S5: Expression analysis of the key autophagy markers LC3-II and p62 indicates higher autophagy levels in cHL than in B-NHL and normal tissue

Immunohistochemistry score	0 (% cases)	1 (% cases)
**LC3-II HRS** cells	3/17 (18)	14/17 (82)
**LC3-II** reactive infiltrate	17/17 (100)	0/17 (0)
**LC3-II control tissue**	3/3 (100)	0/3 (0)
**LC3-II** in BL cells	4/4 (100)	0/4 (0)
**p62 HRS** cells	14/17 (0)	3/17 (18)
**p62** reactive infiltrate	11/17 (65)	6/17 (35)
**p62 control tissue**	1/3 (33)	2/3 (67)
**p62** in BL cells	1/4 (25)	3/4 (75)

Immunohistochemical stainings were performed of a series of 17 cHL cases, 4 BL cases and 3 cases of normal lymphoid tissue for the key autophagy markers. Immunohistochemistry score of tumor cells (Hodgkin Reed-Sternberg (HRS) cells) was analyzed as percentage of cHL cases, BL cases and control tissue; score 0 = negative staining (<50% cells), score 1 = positive (<50% cells). The stainings were evaluated independently by two pathologists without knowledge of other findings.
