# Supplementary material for: Basal autophagy is pivotal for Hodgkin and Reed-Sternberg cells' survival and growth revealing a new strategy for Hodgkin lymphoma treatment
Source: Oncotarget. 2016 Jun 27;7(29):46579–88. doi: 10.18632/oncotarget.10300 (PMC5216819; doi:10.18632/oncotarget.10300)
Supplement: Supplementary file 2 [file oncotarget-07-46579-s002.doc]

Immunohistochemistry score 0 (% cases) 1 (% cases)

**Beclin-1** **HRS** cells 5/17 (30) 12/17 (70)

Beclin-1 reactive infiltrate 17/17 (100) 0/17 (0)

**Beclin-1 BL tissue** 3/3 (100) 0/3 (0)

**Beclin-1 control tissue** 3/3 (100) 0/3 (0)

**Lamp1** **HRS** cells 2/17 (12) 15/17 (88)

Lamp1 reactive infiltrate 15/17 (88) 2/17 (12)

**Lamp1 BL tissue** 3/3 (100) 0/3 (0)

**Lamp1 control tissue** 3/3 (100) 0/3 (0)

**Lamp2** **HRS** cells 2/17 (12) 15/17 (88)

Lamp2 reactive infiltrate 15/17 (88) 2/17 (12)

**Lamp2 BL tissue**  3/3 (100) 0/3 (0)

**Lamp2 control tissue** 3/3 (100) 0/3 (0)

**PINK1** **HRS** cells 3/17 (18) 14/17 (82)

PINK1 reactive infiltrate 15/17 (88) 2/17 (12)

**PINK1 BL tissue** 3/3 (100) 0/3 (0)

**PINK1 control tissue** 3/3 (100) 0/3 (0)

**Park2** **HRS** cells 2/17 (12) 15/17 (88)

Park2 reactive infiltrate 15/17 (88) 2/17 (12)

**Park2 BL tissue** 3/3 (100) 0/3 (0)

**Park2 control tissue** 3/3 (100) 0/3 (0)

**ULK1** **HRS** cells 4/17 (24) 13/17 (76)

ULK1 reactive infiltrate 15/17 (88) 2/17 (12)

**ULK1 BL tissue**  3/3 (100) 0/3 (0)

**ULK1 control tissue** 3/3 (100) 0/3 (0)

**ULK2** **HRS** cells 1/17 (6) 16/17 (94)

ULK2 reactive infiltrate 17/17 (100) 0/17 (0)

**ULK2 BL tissue** 3/3 (100) 0/3 (0)

**ULK2 control tissue** 3/3 (100) 0/3 (0)
